# Development of supermale and all-male Atlantic salmon to research the *vgll3* allele - puberty link

**DOI:** 10.1186/s12863-020-00927-2

**Published:** 2020-11-12

**Authors:** Per Gunnar Fjelldal, Tom J. Hansen, Anna Wargelius, Fernando Ayllon, Kevin A. Glover, Rüdiger W. Schulz, Thomas W. K. Fraser

**Affiliations:** 1 Institute of Marine Research (IMR), Matre Aquaculture Research Station, 5984 Matredal, Norway; 2 Institute of Marine Research (IMR), PO Box 1870, Nordnes, 5817 Bergen, Norway; 3 Reproductive Biology Group, Division Developmental Biology, Department of Biology, Faculty of Sciences, Utrecht University, Utrecht, The Netherlands

**Keywords:** Atlantic salmon, All-male, Double haploid, YY supermale, Maturation, Puberty, vgll3

## Abstract

**Background:**

Farmed Atlantic salmon are one of the most economically significant global aquaculture products. Early sexual maturation of farmed males represents a significant challenge to this industry and has been linked with the *vgll3* genotype. However, tools to aid research of this topic, such as all-male and clonal fish, are still lacking. The present 6-year study examined if all-male production is possible in Atlantic salmon, a species with heteromorphic sex chromosomes (males being XY, females XX), and if all-male fish can be applied to further explore the vgll3 contribution on the likelihood of early maturation.

**Results:**

Estrogen treatment of mixed sex yolk sac larvae gave rise to one sexually mature hermaphrodite with a male genotype (XY) that was used to produce both self-fertilized offspring and androgenetic double haploid (dh) offspring following egg activation with UV treated sperm and pressure shock to block the first mitotic division. There were YY supermales among both offspring types, which were crossed with dh females. Between 1 and 8% of the putative all-male offspring from the eight crosses with self-fertilized supermales were found to have ovaries, and 95% of these phenotypic females were also genetically female. None of the offspring from the one dh supermale cross had ovaries. When assessing the general contribution of the *vgll3* locus on the likelihood of early post-smolt sexual maturation (jacking) in the all-male populations we found individuals that were homozygous for the early maturing genotype (97%) were more likely to enter puberty than individuals that were homozygous for the late maturing genotype (26%). However, the likelihood of jacking within individuals with an early/late heterozygous genotype was higher when the early allele came from the dam (94%) compared to the sire (45%).

**Conclusions:**

The present results show that supermale Atlantic salmon are viable and fertile and can be used as a research tool to study important aspects of sexual maturation, such as to further explore the sex dependent parental genetic contribution to age at puberty in Atlantic salmon. In addition, we report the production of viable double haploid supermale fish.

**Supplementary Information:**

The online version contains supplementary material available at 10.1186/s12863-020-00927-2.

## Background

Aquaculture continues to expand rapidly on a global basis and is regarded as an important future source of protein production to feed the ever-growing human population. Within this food-production sector, Atlantic salmon (*Salmo salar L.*) represents one of the most highly domesticated [1] and economically significant species [2], accounting for approximately 2.6 million tonnes of production in 2019. Market sized-salmon are typically produced in open sea-cages and are therefore exposed to the natural elements that influence the salmon’s biology. One of the most significant and persistent challenges in this regard is that of early sexual maturation, especially in males [3].

Early sexual maturation of male Atlantic salmon in aquaculture is regarded as a major problem [3–5] because it affects health and welfare, growth [6, 7], and down-grading losses at harvest [6]. During primary processing, mature fish are either sorted as low-quality grade, with reduced price, or discharged, depending on maturity status, i.e. if the fish are maturing or fully mature. When kept in stimulatory rearing environments, sexual maturity rates in domesticated male Atlantic salmon (*Salmo salar*) postsmolts may exceed 80% [8–10] during the early seawater phase when the fish are around 500 g, and the problem is primarily caused by the use of elevated rearing temperature together with photoperiod manipulation [9, 11, 12] . Artificial spring/summer like conditions of warm environment combined with a short day to continuous light switch are used to enhance early life growth and/or induce the parr-smolt transformation, the process by which salmon alter their physiology before transition from freshwater to seawater. However, these same conditions are also factors triggering precocious puberty [9, 11]. Mature postsmolts, also known as jacks, show depressed growth [12] and are a source for reduced animal welfare due to compromised health [3] and reduced hypo-osmoregulatory ability [10]. Furthermore, sexual maturity leads to downgrading losses during primary processing due to reduced flesh quality or secondary sexual characteristics that can be partly retained even after the fish revert back to an immature state, particularly the shape of the head and the scale formation [13]. Therefore, there is a need to develop strategies for reducing pre-harvest sexual maturation in Atlantic salmon.

Photoperiod manipulation can be used to reduce the levels of postsmolt maturation, but this method is not 100% effective [11]. Age of puberty in Atlantic salmon is known to be heritable [6]. Most notably, Ayllon et al. [14] and Barson et al. [15] showed that the *vgll3* locus located on chr 25 accounts for approximately 33–36% of the variation in the age of sexual maturity in wild and/or domestic male salmon that have experienced one or more winters in seawater. The *vgll3* locus has also been found to explain 21% of the variation in the prevalence of wild mature male parr, also known as sneaker males, to complete sexual maturation at a small body size and delay migration to seawater [16]. In farmed salmon, fish carrying the late maturity *vgll3* variant (LL) mature later than those with the early maturity variant (EE), with those heterozygous for *vgll3* (EL) being intermediate [17]. In addition, the *vgll3* genotype has also been found to affect size at maturity in wild males [15], with LL being 25% larger than EE males when maturing at the same age. How genetic variation and maternal and paternal contribution in this locus impacts on jacking and/or growth is, however, unknown.

**Fig. 1:**
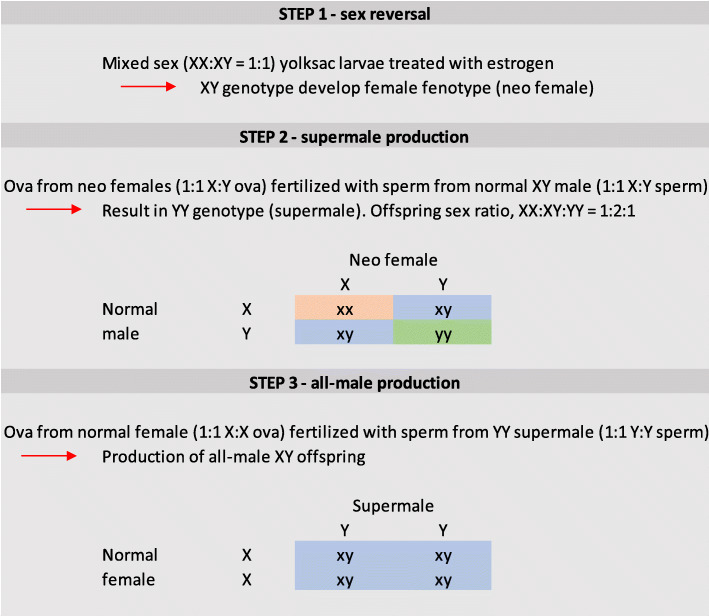
Schematic showing the generation of neo-female, supermale and all-male populations

In Atlantic salmon, sex is genetically determined via a master sex-determining gene, sdY (XX female, XY male, [18]) with a sex ratio of 1:1. Hence, all-male populations would be an effective tool to half the number of experimental animals needed to research early maturity in males. To produce all-male offspring in female homogametic species, such as salmonids [19–21], YY supermales are needed. Although Atlantic salmon supermales have never been produced, this has previously been achieved in several other teleost species [reviewed by [22]]. To produce salmonid YY supermales, one can first subject genetic males to estrogen [23, 24], leading to sex-reversal and the production of neo-females. Neo-females produce X and Y eggs that when fertilized with X and Y sperm give 25% YY, 50% XY, and 25% XX offspring (Fig. 1).

A further reduction in the use of experimental fish in Atlantic salmon research could be accomplished by the production of genetically standardized fish, since research on animals with unknown or variable genetic constitution increase the number of animals needed to produce significant results and genetically standardized fish will increase reproducibility [25]. To achieve this, one can use gynogenesis to produce diploid (double haploid) individuals whereby the eggs are activated with UV irradiated sperm and a pressure shock is used to prevent the first mitotic division [26–28]. This results in the creation of an individual that maintains both the original maternal chromosome sets without any contribution from the male. Recently Hansen et al. [29] optimized the protocols for sperm inactivation with UV light and timing of hydrostatic pressure to produce meiotic diploid gynogenesis and developed a method to produce gynogenetic double haploid Atlantic salmon as founders for isogenic lines.

The present study on Atlantic salmon was designed in order to test, (i) if neo-female production is possible, (ii) if neo-females produce viable YY supermale off-spring, and (iii) if crosses between YY supermales and double haploid females with different vgll3 genotypes produce all-male off-spring that can be used to explore the vgll3 contribution on the likelihood of jacking. For this purpose, we sex-reversed genetic males (neo-females), with different vgll3 genotypes, to produce supermales, made double haploid females with different vgll3 genotypes which were crossed with the supermales to produce all-male offspring, and finally subjected these all-male populations to an environmental regime know to stimulate jacking.

## Results

### Sex reversal – the Golden fish

**Fig. 2:**
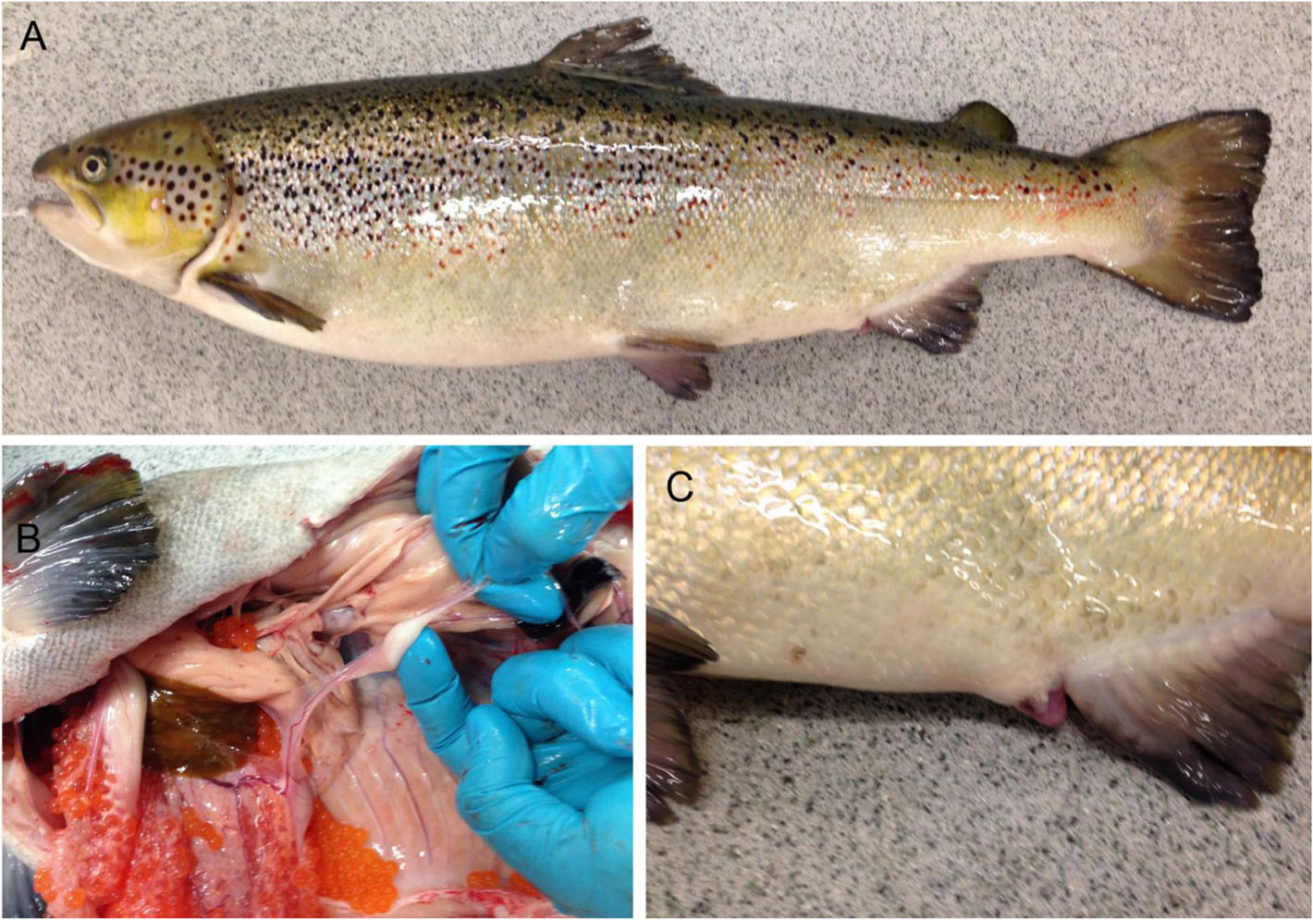
Photographs of the Golden Fish. **a** Whole fish – female phenotype. **b** Running milt and ovulated eggs – hermaphrodite. **c** Gonopore – female phenotype

Following sex reversal with ethynylestradiol-17, only one fish (hereafter called the ‘Golden Fish’) had a mismatch between genetic and phenotypic sex at sexual maturation. The Golden Fish had a female phenotype (Fig. 2a,c), both running milt and ovulated eggs (hermaphrodite, Fig. 2b), but was genetically male (XY), heterozygous for *vgll3* (EL), and displayed typical genetic variation among the 18 markers tested (Supplementary Table 1).

### Production of YY supermales

During stripping of the Golden Fish, both milt and ova were released, resulting in part of the eggs being self-fertilized. The remaining ova were carefully dissected out. These were presumably un-fertilized, since there were no macroscopic traces of milt inside the abdomen. The dissected ova were fertilized with UV irradiated sperm and subjected to hydrostatic pressure for double haploid (dh) production. When screening for mature supermale parr among the Golden Fish off-spring, two mature supermales (sire 1 and 2) were found among the self-fertilized offspring, and one mature supermale (sire 3) among the dh offspring. Sires 1 and 2 were heterozygous for *vgll3* (EL) and had a double sdY dose and were therefore considered to have a supermale – YY – genotype. Genetic variation was lower in sire 1 and 2 when compared to their parent, the Golden Fish (Supplementary Table 1). The Golden Fish was homozygous on 27% of the tested markers, while sires 1 and 2 were homozygous on 64 and 52% of the tested markers (Supplementary Table 1), respectively. The genetic sex ratio of the progeny from the self-fertilization followed a perfect Mendelian distribution, with 25% YY, 50% XY, and 25% XX individuals.

The dh-YY supermale (sire 3) was homozygous for the early maturing vgll3 genotype (EE) and had a double sdY dose and supermale – YY – genotype. There was no genetic variation in sire 3; it displayed an identical single allele for each of the 18 markers tested (Supplementary Table 1). The sex ratio of the progeny from the dh production was 54.5% YY and 45.5% XX. This confirmed the assumption that the dissected ova were un-fertilized.

### Production of double haploid females

The five selected vgll3 homozygous dh sibling females (dams 1–5), three with early maturing genotype (EE, dam 1, 2 and 5) and two with late (LL, dam 3 and 4), showed no genetic variation; all animals displayed an identical single allele for each of the 18 markers tested (Supplementary Table 1).

### All-male production

Ova from sibling dams 1–4 were each split in two equal parts and fertilized with milt from sire 1 or 2, creating two half sibling groups per dam, while the ova from dam 5 were fertilized with milt from sire 3. This created totally 9 different family groups (Table 1). The proportion of males in these groups varied between 92 and 100%. Of the 44 phenotypic females, the DNA was available for 40 individuals of which 2 (5%) were confirmed genetically male (sdY-positive), the others being genetically female (sdY-negative).

**Table 1:** Occurrence of phenotypic females in the putative all-male populations

Dam	1		2		3		4		5
Sire	1	2	1	2	1	2	1	2	3
Male offspring (N)	131	121	169	179	163	175	172	176	89
Female offspring (N)	6	3	8	1	14	3	6	3	0
% males	95.6	97.6	95.5	99.4	92.1	98.3	96.6	98.3	100.0

### General effects of vgll3

In support of our hypothesis, when the data was pooled across all families there was a significant effect of vgll3 genotype (Table 2) on the likelihood of jacking (EE > EL > LL), body mass at the start of the experiment (EE > EL > LL), and body condition at the start of the experiment (EE > EL > LL), but, there was no effect on GSI within jacks. However, these genotype effects on pooled data did not hold true for body mass, body condition, or GSI when correcting for family (see below).

**Table 2:** Results from models looking at genotype effects on maturity status at the end of the experiment and body size parameters at the beginning of the experiment. Note, the null model had a better fit than genotype for GSI

Parameter	Genotype			Statistics					
	EE	EL	LL	Model	χ^**2**^	df	p	R^**2m**^	R^**2c**^
Jacking (odds ratio)	0.97 (0.95–0.99)^a^	0.70 (0.66–0.74)^b^	0.26 (0.21–0.32)^c^	GLMER (binomial)	249	2	< 0.001	0.47	0.47
GSI (% body mass) - jacks only	1.94 (1.86–2.02)	1.86 (1.79–1.94)	1.86 (1.68–2.04)	LME	–	–	ns	–	–
Body mass (g) - day 0	128 (124–132)^a^	116 (113–119)^b^	108 (104–112)^c^	LME (log)	119	2	< 0.001	0.08	0.08
Body condition (*K* factor) - day 0	1.24 (1.23–1.26)^a^	1.24 (1.22–1.25)^b^	1.23 (1.21–1.24)^c^	LME (log)	24	2	< 0.001	0.02	0.06

Different lowercase letters indicate significant differences between genotypes (Post hoc. Least square means, *p* < 0.05)

### Parental effects in EE vs EL and EL vs LL

The percentage of jacks within all families maintained the ranking of the general model (EE > EL > LL) with the exception of one EE dam × EL sire cross for which all progeny entered puberty irrespective of genotype (Supplementary Table 2). Models that controlled for family on the likelihood of jacking suggested dam and sire effects explained more of the variation than *vgll3* (Table 3), although *vgll3* was still significant when averaging over dam and sire effects (Table 4).

**Table 3:** Results from models looking for family effects with genotype. Fixed effects with a *p* value > 0.2 are not shown for clarity, nor lower order fixed effects that are involved in an interaction with a *p* value < 0.05

Comparison	Parameter	Model		R^**2m**^	R^**2c**^	Highest order of significance	χ^**2**^	df	p	
EE vs EL	Jacking	GLM (binomial)	Genotype + dam + sire	0.39	–	Genotype	19.0	1	< 0.001	***
						Sire	38.7	1	< 0.001	***
	GSI in pubertal fish	LME	Genotype × dam × sire	0.44	0.44	Genotype × Sire	4.3	1	0.039	*
						Genotype × Dam	2.4	1	0.123	
						Dam × Sire	9.7	1	0.002	**
	Body mass - day 0	LME (log)	Genotype × dam × sire	0.09	0.09	Dam × Sire	10.5	1	0.001	**
						Genotype × Dam	5.2	1	0.022	*
	Body condition - day 0	LME (log)	Genotype × dam × sire	0.20	0.28	Dam	63.0	1	< 0.001	***
						Sire	7.7	1	0.005	**
						Dam × Sire	2.7	1	0.098	
EL vs LL	Jacking	GLMER (binomial)	Genotype × dam × sire	0.15	0.16	Dam	9.2	1	0.002	**
						Sire	6.0	1	0.014	*
	GSI in pubertal fish	LME	Genotype × dam × sire	0.77	0.79	Genotype × Dam × Sire	5.1	1	0.024	*
	Body mass - day 0	LME	Genotype × dam × sire	0.03	0.03	Dam	6.5	1	0.011	*
						Sire	2.9	1	0.086	
	Body condition - day 0	LME	Genotype × dam × sire	0.06	0.07	Dam × Sire	10.9	1	< 0.001	***
EL	Jacking	GLM (binomial)	Dam + sire	0.42	–	Dam	183.6	3	< 0.001	***
						Sire	44.1	1	< 0.001	***
	GSI in pubertal fish	LME	Dam × sire	0.39	0.39	Dam × sire	16.8	3	< 0.001	***
	Body mass (g) - day 0	LME (log)	Dam × sire	0.19	0.21	Dam × sire	11.3	3	0.010	*
	Body condition (K factor) - day 0	LME	Dam × sire	0.19	0.21	Dam × sire	12.9	3	0.005	**

*** *p* < 0.001, ** *p* < 0.01, * *p* < 0.05

**Table 4:** Results from lsmeans tests looking at genotype effects when accounting for dam and sire effects. The main model results can be found in Table 3. The data presented are lsmeans (lower and upper confidence intervals)

Parameter	EE	EL	LL	Estimate/ratio	SE	df	z/t ratio	***p***	
Jacking (odds ratio)	0.986 (0.970–0.994)	0.939 (0.900–0.964)	–	4.6	1.80	Inf	3.93	< 0.001	***
GSI (% body mass) - jacks only	1.84 (1.78–1.91)	1.71 (1.65–1.78)	–	0.1	0.03	636	4.01	< 0.001	***
Body mass (g) - day 0	120 (117–123)	123 (119–126)	–	1.0	0.01	681	−1.70	0.090	
Body condition (K factor) - day 0	1.24 (1.23–1.26)	1.24 (1.23–1.26)	–	1.0	0.003	681	−0.09	0.930	
Jacking (odds ratio)	–	0.450 (0.386–0.516)	0.246 (0.195–0.306)	2.5	0.49	Inf	4.69	< 0.001	***
GSI (% body mass) - jacks only	–	1.99 (1.85–2.13)	1.78 (1.63–1.94)	0.2	0.07	201	2.76	0.006	**
Body mass (g) - day 0	–	110 (107–113)	109 (106–113)	0.4	1.79	569	0.23	0.819	
Body condition (*K* factor) - day 0	–	1.23 (1.22–1.24)	1.23 (1.22–1.24)	0.003	0.004	569	0.65	0.513	

*** *p* < 0.001, ** *p* < 0.01, * *p* < 0.05

In contrast to the general model (i.e. data was pooled for family), we found genotype effects on the GSI of jacks. In brief, there was a 2-way interaction between genotype and sire in the EE vs EL model and a significant 3-way interaction between genotype, dam, and sire in the EL vs LL model (Table 3). Although the differences were not always significant within families the trends were always the same (EE > EL and EL > LL, Supplementary Table 2), therefore when data was averaged over dam and sire effects EE had higher GSI values than EL and EL had higher GSI values than LL (Table 4).

In contrast to the general model, genotype had no effect on body mass or condition between EE and EL or EL and LL males on day 0 (Table 4). However, dam and sire effects were apparent.

### Parental effects in EL fish only

**Fig. 3:**
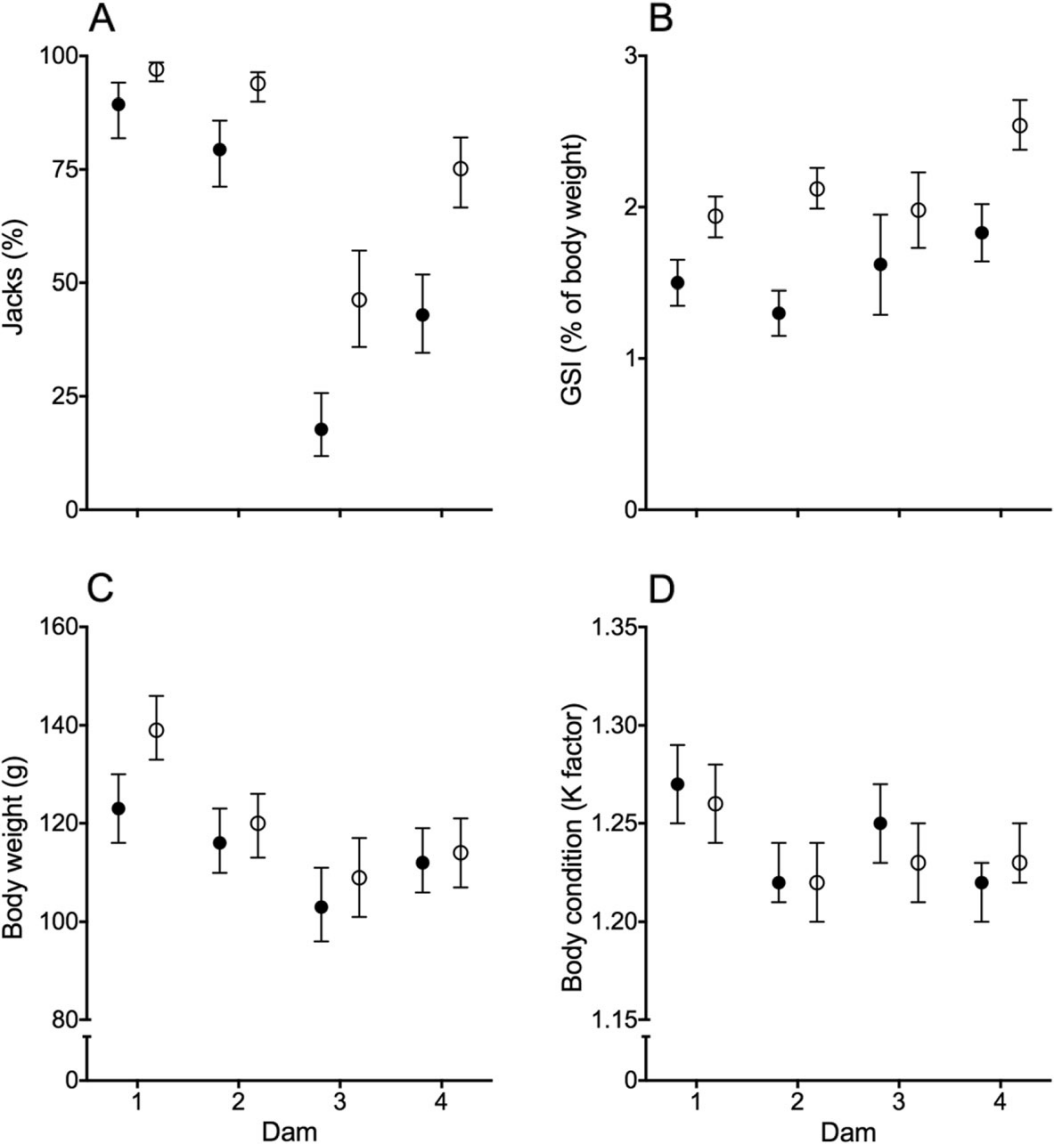
Dam and sire effects on maturity status at the end of the experiment and body size and condition at the start of the experiment. Data are means +/− 95% CI

Significant dam and sire effects were observed on the percentage of jacks produced by EL males (Table 3). Sire 2 had a higher odd ratio of producing jacks than sire 1 and there was also a strong dam effect with dam 1 = 2 > 3 > 4 (Fig. 3a). Here, it is noted that dams 1 and 2 were both EE whereas dams 3 and 4 were LL. There was also a significant dam × sire interaction on GSI in jacks. Here, the offspring of sire 2 always had a higher GSI than the offspring of sire 1 when crossed with the same dam, but the order of the dam effect was dependent on the sire (Fig. 3b). Prior to the maturation stimulating regime, dam and sire effects were also observed on body mass and condition. The ranking of body mass for each dam and sire matched the jacking results (Fig. 3c), but not so for body condition (Fig. 3d).

## Discussion

This study documents the first successful production of all-male Atlantic salmon. This was achieved in several steps, first by production of a sexually mature hermaphrodite with a male genotype (XY) that was used to produce both self-fertilized and double haploid (dh) YY offspring, and then by crossing these YY supermales with dh females. Having achieved this, we then used the resulting all-male population to investigate the contribution of the *vgll3* genotype to postsmolt maturation (jacking). *Vgll3* EE individuals had higher prevalence’s of jacking than LL individuals when exposed to environmental conditions that stimulate puberty in smolts. In addition, we found strong paternal and maternal effects on the prevalence of jacking independent of *vgll3*.

### Neo-female and supermale production

In the present study we managed to produce one hermaphroditic Atlantic salmon with male genotype and female phenotype, who produced viable sperm and eggs. It is unclear why the success of the sex-reversal was so low as Piferrer and Donaldson [30] reported 100% success following the same protocol in chinook salmon (*Oncorhynchus tshawytscha*) and a slightly better success than with use of Estradiol-17β, and Johnstone et al. [31] reports 100% feminisation in Atlantic salmon after feeding Estradiol-17β during first feeding. With this background it is obvious that the protocol could be optimized to increase the success of sex-reversal. Similar to our findings, self-fertilization of mature hermaphrodites has been recorded in estrogen treated rainbow trout, with 76.6% male offspring, suggesting viability of the YY genotype [24]. The YY genotype was confirmed among the offspring of the Golden Fish using a recently developed qPCR method [32], which confirmed 25% YY, 50% XY and 25% XX genotype distribution among the offspring from the self-fertilization. Thus, the Golden Fish had a 1:1 ratio of X and Y eggs and sperm. When applying the method described to produce Dh females (dh-XX) [29] on the eggs of the Golden Fish, we were able to produce Dh YY supermale and Dh female (XX) off-spring with a sex ratio of approx. 1:1. To our knowledge, this is the first report of production of Dh YY fish.

### Occurrence of females in putative all-male populations

A portion (1–8%) of the all-male progeny developed ovaries, with 5% of these having a XY genotype, and 95% a XX genotype. Indeed, in rainbow trout, several studies on supposed male or female mono-sex populations have shown a low frequency of individuals displaying the opposite sex phenotype than the supposed sex. For instance, there are reports of 2% males among meiotic gynogens [33], 1–6% males among offspring of assumed XX males [34], and of 1% females among offspring of assumed supermales [24]. Unexpected maleness among mitotic gynogens has been attributed to specific recessive mutations in rainbow trout (termed *mal* mutation) [35] and carp (termed *mas* mutation) [36], and the occurrence of occasional females in the progeny of YY males have been suggested to have a genetic basis in Nile tilapia (*Oreochromis niloticus*) [37]. Furthermore, environmental factors, especially temperature, may also influence sex differentiation in fish (reviewed by [38]). This is linked to increased glucocorticoid levels under stressful conditions that can override genetic sex determination mechanisms (review [39]). Valdivia et al. [40] studied the effect of temperature on masculinization rate in all-female rainbow trout populations that carried the mal mutation [35] and found a 2-fold increase in masculinization rate at high temperature and a strong impact of genetic background. Our fish populations were reared under stable and moderate temperature, suggesting a genetic origin behind the occurrence of females among supermale offspring. That 5% of the females were genetically males is interesting, since these could potentially produce YY offspring if crossed with normal XY males.

### Genotype and the likelihood of jacking

Subjecting our all-male progeny to a maturation stimulating regime with a shift from short day to continuous light and 16 °C induced puberty in all 9 families. The mean GSI values in maturing males after 8 weeks under these stimulatory conditions ranged between 1.29 and 2.54, values that concur with earlier studies [11]. As expected, based on previous work in parr [16] and sea-migrating males [14, 15], the EE fish had the highest incidences of puberty whereas the LL fish had the lowest. EE males also showed a higher likelihood of maturation than EL males, which is in contrast to the finding of Barson et al. [15] in wild fish, but similar to previous work in farmed males [17]. As such, it appears *vgll3* could be targeted in domestic breeding programs to reduce both the incidence of jacking and grilsing (maturation after 1 sea-winter). However, why the E allele does not appear to be dominant to the L allele in farmed vs. wild males is currently unknown.

Although the *vgll3* genotype had a significant effect on the likelihood of entering puberty, dam and sire effects were apparent that were not explained by *vgll3*. This suggests that other areas of the genome are of importance when explaining the likelihood of jacking, not only *vgll3*. Previous studies have found areas of the genome other than that identified on chromosome 25 can be associated with sea age at maturation [41] whilst others have suggested *vgll3* may work in synergy with other genes such as TEAD3 [42] and *six6* [15]. In a study on *six6* and *vgll3* associations with age at maturity in four species of Pacific salmon, Walters et al. 2020 [43] found a significant association between *six6* and age at maturity in two species, but not for *vgll3* in any species. Further work is required in order to understand the interplay between these genes in Atlantic salmon.

Current theory suggests there is an energy threshold at a given size required in order to enter puberty in salmon [3, 44]. Indeed, across all families, fish that went on to become jacks had significantly higher mean weights than those that remained immature. However, there was no difference in body condition, a proxy for energy reserves [45]. Although there was a general association between body mass and *vgll3* genotype, this did not hold true following within family analyses. Instead, similar to the likelihood of jacking, dam and sire effects were more influential. Therefore, although body size does appear to be an important predictor for jacking and was generally associated with genotype, body size was not explained by genotype within family.

Previous work in wild fish found mature LL salmon to be larger (e.g. 25% larger in 3-seawinter males) than age-matched mature EE conspecifics, although growth prior to maturation was not presented [15]. In the current study, we found the opposite with a general tendency for EE > EL > LL in immature males when pooling all data, but there was no genotype effect on body weight within families. The contrasts in background material between our work and that of Barson et al. [15] are considerable, with large differences in genetic background, life stage comparison, and rearing environment, which may contribute to this discrepancy. Nevertheless, we found that pubertal EE males had higher GSIs than EL males, and in turn, EL males had higher GSIs than LL males. It is not clear if these differences are explained by either the timing of puberty, with one genotype initiating puberty earlier than the other, or the speed of development, with one developing at a quicker rate than the other. However, if EE males do continue to develop larger gonads than LL individuals this would likely come at a greater somatic cost as puberty is an energetically demanding process. Therefore, as we sampled fish relatively early in pubertal development, future work could assess somatic and gonad growth over the entire maturation cycle to see if genotype effects emerge further along in the process and whether these are linked to body size at maturation.

Although the present study has a limited number of families when comparing parental effects within genotype, it was noted that the EL progeny that attained the E allele from the dam had a higher likelihood of jacking than those that received the E allele from the sire. Alternatively, it may also be the other way around, those EL males that received the L allele from the sire were more prone to jacking than those receiving the L allele from the mother. Here, it would be interesting to know whether this holds true in a larger dataset with a higher number of families and whether paternal or maternal epigenetic mechanisms play a role in the age of puberty.

### Implications for breeding and commercial aquaculture

Current Atlantic salmon production mainly relies on the production of mixed sex stocks even though males have both production and environment related advantages over females. For example, even though Atlantic salmon are considered sexually monomorphic prior to sexual maturation [7] males have been found to be heavier than females among immature individuals [46–48]. In addition, males are less likely to genetically introgress with wild populations of salmon if they escape from the farm, compared to females [49]. However, despite these considerable advantages, all-male stocks are not in use for two reasons. Firstly, the YY broodstock that is required to make all-male offspring are not commercially available. Secondly, males have a higher propensity to sexually mature prior to harvest size than females, and sexual maturity is associated with several negative traits including reduced somatic growth, poor flesh quality, reduced animal welfare, and an increased risk of disease [3]. The current study shows that production of YY broodstock is possible in Atlantic salmon and that selecting broodstock with a certain genotype could potentially contribute to solving problems associated with sexual maturation.

Jack and grilse (1 sea-winter) maturation are the most problematic maturation phenotypes in the culture of male Atlantic salmon today. This is especially true given that an ever-increasing amount of today’s production has turned to recirculation aquaculture system (RAS) technology in order to produce smolts, and these systems rely on constant elevated water temperatures which are known to stimulate male puberty [5]. This is common for global Atlantic salmon production, whereas the environment in sea-cages is seasonally variable and region specific. As such, applying all-male stocks in sea-cage farming in the colder areas, such as Northern Norway, where the grilsing rate is generally low, could potentially reduce production time without compromising flesh quality and fish welfare. However, the interaction with production method on land and genotype needs to be addressed before further advice can be given.

## Conclusions

The study showed that double haploid and self-fertilized YY supermale Atlantic salmon were viable and fertile and gave all-male offspring, in which a strong relationship between vgll3 genotype and likelihood of jacking was observed. This achievement provides a significant new research tool and can potentially have a major impact on Atlantic salmon aquaculture since males grow faster than females.

## Methods

**Fig. 4:**
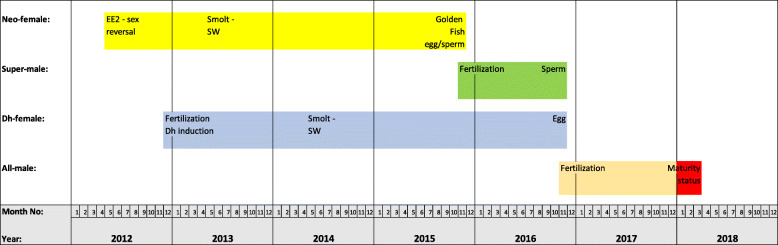
The timeline for production of the different fish groups used in the current study. The period marked with red is the period where the all-male fish were reared under continuous light and 16C to stimulate jacking

All experiments were done with eggs and milt from the domesticated and commercially available Aquagen strain, Aqua Gen AS, Trondheim, Norway. Figure 4 shows the timeline for production of the different fish groups used in the current study.

### Genotyping – genetic background/variation, sex and vgll3

To determine the genetic variation in the Golden Fish, sires 1–3, and dams 1–5, eighteen microsatellite DNA markers were genotyped using standard isolation and amplification protocols previously described in detail [50, 51].

Genotyping for sex served to distinguish potentially successfully sex-reversed fish (exposed to EE2) and distinguish YY from XY males. Total DNA was purified from whole adipose fins using Qiagen DNeasy Blood & Tissue Kit (Qiagen, Hilden, Germany) according to the manufacturer’s recommendations. Sex was validated by a PCR-based method aimed to detect the presence of the sdY gene [18]. Individuals showing amplicons of exon 2 and 4 were designated as males. As a positive PCR control, and for species determination, we used the presence of the 5SrRNA gene [52]. PCR amplifications were performed using reaction mixtures containing approximately 50 ng of extracted Atlantic salmon DNA, 10 nM Tris–HCl pH 8.8, 1.5 mM MgCl_2_, 50 mM KCl, 0.1% Triton X-100, 0.35 μM of each primers, 0.5 Units of DNATaq Polymerase (Promega, Madison, WI, USA) and 250 μM of each dNTP in a final volume of 20 μL. PCR products were visualized in 3% agarose gels. For distinguishing between XY and YY males we used the method recently described by [32].

Genotyping of the *vgll3* locus was performed using allelic discrimination assay for the two missense SNPs in vgll3 according to [14] and served to distinguish three different genotypes: (1) homozygous early (EE), (2) homozygous late (LL), and (3) heterozygous early/late (EL).

### Sex reversal

On the 23rd May 2012, newly hatched alvelins (out bred from the commercially available and highly domesticated Aquagen strain) were immersed in a water bath for 2 h in 400 μg/L ethynylestradiol-17α (EE2) in 0.04% EtOH [30]. The bath treatment was applied 3 days after 50% of the embryos hatched. The fish were first fed in one 1 m ø tank under continuous light and 12 °C. On the 10th December 2012, fish were transferred to one 1.5 m ø tank. The temperature was changed to natural temperature on the 21st June 2012 and photoperiod changed from continuous to natural on the 1st October 2012. The water was changed from fresh- to salt-water on the 8th May 2013. The seawater temperature was stable at 9 °C and the photoperiod was simulated natural (60^o^ N). On the 12th February 2014, all fish were tagged with an electronic transponder for individual recognition (i.e. PIT tag), had the adipose fin removed (stored in ethanol) for DNA extraction and genotyping, and transferred to one 5 × 5 m (7 m deep) sea-cage with natural photoperiod. Fish were genotyped for sex, *vgll3*, and genetic variation (microsatellites).

After 916 days in seawater, and 636 days in the sea-cage, on the 10th November 2015, all mature fish were checked for the relationship between genetic sex and external phenotype. Only one fish (hereafter called the ‘Golden Fish’) had a mismatch, with a male (XY) genotype, but a female phenotype. The Golden Fish was heterozygous for the early (E) and late (L) maturing *vgll3* genotype (EL). Thus, this fish should produce X and Y eggs of both the E and L maturing genotype.

### Production of YY supermales

Upon stripping the Golden Fish (killed by an overdose of anaesthetic; Finquel vet. 0.5 g L^− 1^), both milt and ova were released as it turned out to be a hermaphrodite. Subsequently, we first put the self-fertilized (self) eggs into an incubation tray and then gently dissected out the remaining ova to avoid further self-fertilization. There were no traces of milt inside the abdomen – the fish must have had a functional sperm duct – and the dissected ova were presumably un-fertilized. In order to produce double haploid (dh) offspring from the Golden Fish, the surgically removed eggs were fertilized with UV-irradiated milt, incubated at 8 °C for 4700 minC, subjected to a hydrostatic pressure of 655 bar for 5 mins (TRC-APV, Aqua Pressure Vessel, TRC Hydraulics inc., Dieppe, Canada) [29], and then transferred to an incubation tray. Eggs were incubated at 6 °C. Fish were first fed under a light dark (LD) regime of 12:12 and 12 °C in order to induce parr maturation in males [53]. Fish were pit-tagged and genotyped for sex, *vgll3*, and genetic variation (microsatellites) on the 7th September 2016. It was confirmed that we had YY supermales among the fish, both from the self (self-YY) and dh production (dh-YY). On the 17th November 2016, when grading out fully mature male parr, we found two mature self-YY’s (sire 1 and 2), both EL for *vgll3*, and one mature dh-YY (sire 3), EE for *vgll3* (Supplementary Table 1). Sire 1 and 2 were killed by an overdose of anaesthetic (Finquel vet. 0.5 g L^− 1^), and had their testis dissected and homogenized in Cortland solution (124 nM NaCl, 5.1 mM KCl, 2.9 mM Na_2_HPO_4_). Sire 3 was anesthetized (Finquel vet., 0.1 g L^− 1^), stripped for milt, and kept alive for one more year until December 2017 when it was euthanized (Finquel vet. 0.5 g L^− 1^) for sperm cryopreservation. Sperm from other supermales (not used in the present study) maturing in December 2017 and 2018 was also cryopreserved.

### Production of double haploid females

Dh females (dh-XX) were produced according to the procedure described by Hansen et al. [29]. In brief, on 18 December 2012, ova from six diploid females (Aquagen AS) were mixed and fertilized with UV irradiated sperm and subjected to a high hydrostatic pressure at the first meiotic division. Eggs were incubated following standard production procedures and fish produced as yearling smolts that were transferred to seawater in May 2014. At that stage they were also pit-tagged and tissue sampled. The samples were later genotyped (sex, *vgll3*, and microsatellites). The temperature in seawater was stable at 9 °C and the photoperiod was simulated natural (Western Norway). On the 17th November 2016, the second year in seawater, 5 fully mature ovulated females (dams 1–5**,** Supplementary Table 1) were selected based on *vgll3* genotype, killed by an overdose of anaesthetic (Finquel vet. 0.5 g L^− 1^) and had their eggs stripped. Dams 1, 2, and 5 had the EE *vgll3* genotype, and dams 3 and 4 had the LL *vgll3* genotype. The microsatellite data in Supplementary Table 1 suggests dams 1–5 were all progeny of the same female as they share the same two alleles for all 18 markers. This female must have been *vgll3* heterozygous early/late (EL).

### All-male production

Eggs from dams 1–4 were each split in two equal parts and fertilized with milt from sire 1 or 2, creating two half sibling groups per dam, and a total of 8 different family groups, each with 50/50 occurrence of the two different *vgll3* genotypes since sires 1 and 2 were heterozygous for *vgll3*. Milt from sire 3 was used to fertilize eggs from dam 5.

Each of the 9 family groups (8 from the self-YY (sires 1 and 2) × dh female (dams 1–4) cross, 1 from the dh-YY (sire 3) × dh female (dam 5) cross) were incubated in single trays in a flow-through system at 6 °C. Eggs were mechanically shocked at the eye egg stage on the 9th January 2017 and dead eggs removed. Hatching took place between the 4th and 16th February 2017 and first feeding was on the 22nd March 2017. Each family group were first fed in duplicate start feeding tanks (1 × 1 m, *n* = 18 tanks in total) under continuous light and a stable temperature of 12 °C. The fish were reared in these tanks until the 21st June 2017 when each family group was subsequently transferred to single 3 m tanks (*n* = 9 tanks in total). Here the fish were reared under natural temperature and the photoperiod was changed from continuous light to natural light on 1st October 2017.

### Experimental set up: all-male - vgll3 genotype and jacking

On the 1st December 2017, 180 fish from each of the eight different sire 1 and 2 x dam 1–4 crosses, and 90 from the sire 3 x dam 5 cross, were pit-tagged and distributed in common garden between six 3 m ø tanks, with the same number of individuals from each group in each tank (totally 1530 fish; 255 per tank). Fish were kept under natural light and 6 °C in these tanks until the 8th January 2018 when they were anesthetized (Finquel vet., 0.1 g L^− 1^), had their pit-tag number recorded, measured for fork length and body weight, and moved to six new 3 m ø tanks. On the 9th January 2018, photoperiod was shifted to continuous light and the water temperature was gradually adjusted to 16 °C over a 3-day period to induce maturation [11]. Fish were kept under these conditions until the 6th March 2018, when they were all killed by an overdose of anaesthetic (Finquel vet., 0.5 g L^− 1^), had their pit-tag recorded, were sexed by visual examination of the gonad, and measured for fork length and gonad and body weight, and had their adipose fin sampled (on ethanol) for DNA extraction and *vgll3* genotyping.

### Statistics

Data were transferred to R version 3.6.1 (R Development Core Team 2018, http://www.r-project.org). All the raw data (“vgll3.csv”) and the R script (“vgll3.pdf”) used to analyse the data can be found in the supplementary material. In the analyses described below, the 44 fish that were phenotypic female were excluded. In addition, 4 fish with skeletal deformities were omitted due to their negative effect on growth [54]. One fish had an EE phenotype, even though the dam was LL, and was therefore omitted.

The fish were categorized as immature or pubertal (i.e. jacks) based on GSI. An initial histogram of GSI demonstrated a continuum between 0.01–0.20 and then those > 0.34 (Supplementary Figure 1a). Here, we expect the lower cluster to be immature individuals based on previous studies (immature fish generally have a GSI value of < 0.11: [8, 10, 55]. From previous work we know that in addition to larger testes, jacks have a high growth rate and an increase in body condition during early puberty, above that of immature males [12]. Therefore, we used unsupervised clustering to assess our GSI cut-off. Subsequently, principal component analysis (PCA) using the variables body mass and length at days 0 and 58, and gonad size, confirmed each genotype formed two clusters based on PC1 vs PC2 for which there was no overlap in those fish we had identified as jacks vs immature (Supplementary Figure 1b-d).

Our hypothesis was that EE males would be more likely to mature than LL males, with EL intermediate, following rearing under a maturation stimulating regime (LL and 16C). As the GSI was bimodal, we used a two-step or hurdle model to assess for genotype effects on puberty. The first part of the model assessed the prevalence of pubertal vs immature males within each genotype using a generalised linear mixed modal (GLMER) with a bimodal response, whereas the second part assessed GSI using a linear mixed effect (LME) model depending on genotype within pubertal males only. For the GLMER, jacking (two levels, Y/N) was the dependent variable, genotype (three levels, EE/EL/LL) was set as an independent variable, with tank as a random effect. For the LME, GSI was the dependent variable. Following this, as larger fish with higher energy reserves are expected to mature earlier, we expected EE fish to be the largest fish with the highest body condition and LL fish to be smaller with a lower body condition. To test this hypothesis, we generated linear mixed effect (LME) models with body mass or body condition as dependent variables, genotype (three levels, EE/EL/LL) as the independent categorical variable, and tank as a random effect. Here, we only used body size data from time zero, immediately prior to entering the environmental conditions known to induce puberty.

Following the above general models, the whole analysis was repeated whilst correcting for potential family effects. Here, the cross between dam 5 and sire 3 was not included as they produced only EE offspring. Furthermore, due to the experimental design, parental effects could only be assessed within EL fish, or between EE and EL or LE and LL fish, as dams 1 and 2 and 3 and 4 produced only EE and EL, or LE and LL offspring, respectively. This meant that EE genotypes could not be compared to LL genotypes when assessing family effects within the current experimental design. We then used the same approach as for the general models, but sire and dam were included as categorical independent variables. When comparing for family effects on the prevalence of jacking in the EE vs EL and the EL only comparisons, dam and sire were not allowed to interact, and tank could not be included as a random effect in these models as not all family/maturity types were represented. However, in the EL vs LL comparison and for the analysis of GSI within EE vs EL, EL vs LL and EL fish only, dam and sire were allowed to interact, and tank was included as a random effect.

Model fit was assessed by examination of model residuals (i.e. standardised vs fitted residuals, histograms, and/or q-q plots). Type II sum of squares were used for models without interactions, whereas type III sum of squares were used when interactions were present. The marginal R2 (R^2m^) is reported for all models, using the “r.squareGLMM” command within the “MuMIn” library, and the conditional R2 (R^2C^) is also reported for all models with a random effect. Significance was assigned at *p* < 0.05. Post hoc tests were done using lsmeans within the “emmeans” library.

## Supplementary Information

**Additional file 1: Table S1.** Microsatellite analysis of the Golden Fish, sire 1-3 and dam 1-5. Blank cell = no marker amplification. Vgll3 genotypes are indicated in brackets. **Table S2.** Raw data (mean values) within each family. **Figure S1.** Histogram of testis size (A). PCA plots for EE fish (A), EL fish (B), and LL fish (C) showing that the GSI cut-off of 0.2 leads to the same clustering as when accounting for growth.

## Data Availability

The datasets analysed in the current study are available from the corresponding author on request. Supermale cryopreserved sperm is available.

## Abbreviations

dh: Double haploid
chr: Chromosome
LL: Late/late homozygous
EE: Early/early homozygous
EL: Early/late heterozygous
UV: Ultraviolet
LD: Light dark
PCA: Principal component analysis
GLMER: Generalised linear mixed modal
LME: Linear mixed effect
