# Supplementary material for: Development of supermale and all-male Atlantic salmon to research the vgll3 allele - puberty link
Source: BMC Genet. 2020 Nov 12;21:123. doi: 10.1186/s12863-020-00927-2 (PMC7664053; doi:10.1186/s12863-020-00927-2)
Supplement: Supplementary file 1 — Additional file 1: Table S1. Microsatellite analysis of the Golden Fish, sire 1-3 and dam 1-5. Blank cell = no marker amplification. Vgll3 genotypes are indicated in brackets. Table S2. Raw data (mean values) within each family. Figure S1. Histogram of testis size (A). PCA plots for EE fish (A), EL fish (B), and LL fish (C) showing that the GSI cut-off of 0.2 leads to the same clustering as when accounting for growth. [file 12863_2020_927_MOESM1_ESM.pdf]

Supplementary Table 1. Blank cell = no marker amplification. Vgll3 genotypes are indicated in brackets

Genetic marker	Golden Fish (EL)	sire 1 self-YY (EL)	sire 2 self-YY (EL)	sire 3 dh-YY (EE)	dam 1 dh-XX (EE)	dam 2 dh-XX (EE)	dam 3 dh-XX (LL)	dam 4 dh-XX (LL)	dam 5 dh-XX (EE)
SSsp2201-a	275	283	275	275	299	275	299	299	299
SSsp2201-b	283	283	283	275	299	275	299	299	299
SSsp2210-a	124	124	124	136	148	128	128	148	148
SSsp2210-b	136	136	136	136	148	128	128	148	148
SSspG7-a	127	127	127	127	179	179	147	179	147
SSspG7-b	143	127	143	127	179	179	147	179	147
Ssa202-a	258	258	258	258	242	242	242	242	242
Ssa202-b	258	258	258	258	242	242	242	242	242
SsaD144-a	166	166	198	166	154	186	154	154	154
SsaD144-b	198	198	198	166	154	186	154	154	154
SsaD157-a	343	343	343	343	375	375	343	375	343
SsaD157-b	343	343	343	343	375	375	343	375	343
Sp1605-a	232	232	244	244	224	224	224	216	216
Sp1605-b	244	244	244	244	224	224	224	216	216
Sp2216-a	226	226	226	226	242	258	258	242	258
Sp2216-b	226	226	226	226	242	258	258	242	258
Ssa14-a	140	140	140	140	140	140	144	144	144
Ssa14-b	144	140	144	140	140	140	144	144	144
Ssa171-a	213	241	213	213	233	233	233	217	233
Ssa171-b	241	241	241	213	233	233	233	217	233
Ssa289-a	116				118	118	118	118	118
Ssa289-b	122				118	118	118	118	118
MHC1-a	142	146	142	146	142	136	136	136	142
MHC1-b	146	146	142	146	142	136	136	136	142
MHC2-a	260	260	260	260	370	370	280	280	370
MHC2-b	260	260	260	260	370	370	280	280	370
SSsp3016-a	90	90	90	90	94	130	94	130	94
SSsp3016-b	94	94	94	90	94	130	94	130	94
SsOsl85-a	195	195	195	195	187	193	187	193	193
SsOsl85-b	199	199	199	195	187	193	187	193	193
Ssa197-a	184	184	184	216	188	188	188	184	184
Ssa197-b	216	184	216	216	188	188	188	184	184
SsaD486-a	172	172	172	172	172	172	172	172	172
SsaD486-b	172	172	172	172	172	172	172	172	172
SsaF43-a	115	115	115	121	115	115	115	115	115
SsaF43-b	121	121	115	121	115	115	115	115	115

Supplementary Table 2. Raw data (mean values) within each family.

Parameter	Sire 1 (EL)																Sire 3 (EE)	
	Dam 1 (EE)				Dam 2 (EE)				Dam 3 (LL)				Dam 4 (LL)				Dam 5 (EE)	
	Immature		Jack		Immature		Jack		Immature		Jack		Immature		Jack		Immature	Jack
	EE	EL	EE	EL	EE	EL	EE	EL	EL	LL	EL	LL	EL	LL	EL	LL	EE	EE
n	7	11	92	68	2	21	79	73	51	47	14	8	53	51	44	24	2	85
% (for genotype within family)	7	14	93	86	2	22	98	78	78	85	22	15	55	68	45	32	2	98
GSI	0.05	0.04	1.64	1.50	0.05	0.04	1.29	1.30	0.04	0.04	1.62	1.41	0.05	0.05	1.83	1.80	0.06	2.34
Mass (g) - day 0	85	100	122	127	116	109	122	118	101	102	114	125	99	101	130	124	157	169
Mass (g) - day 58	150	176	333	337	198	194	298	283	173	177	281	302	180	187	341	323	333	434
SGR (% day^{-1}) - day 58	0.96	1.02	1.79	1.73	0.95	1.02	1.57	1.56	0.93	0.94	1.58	1.56	1.07	1.06	1.72	1.69	1.31	1.69
Condition - day 0	1.29	1.28	1.28	1.27	1.23	1.23	1.22	1.22	1.25	1.25	1.26	1.28	1.24	1.22	1.22	1.23	1.17	1.25
Condition - day 58	1.07	1.08	1.32	1.30	1.07	1.07	1.27	1.26	1.07	1.06	1.27	1.29	1.04	1.05	1.31	1.30	1.14	1.36
	Sire 2 (EL)																	
n	0	0	80	89	0	4	78	89	34	53	25	17	21	59	56	26		
%	0	0	100	100	0	4	100	96	58	76	42	24	27	69	73	31		
GSI	-	-	2.26	1.93	-	0.06	2.19	2.12	0.04	0.04	1.98	1.93	0.06	0.05	2.54	2.01		
Mass day 0	-	-	130	139	-	91	117	121	104	106	118	122	106	110	118	124		
Mass day 58	-	-	362	377	-	185	313	312	203	207	309	315	224	225	336	339		
SGR day 58	-	-	1.82	1.77	-	1.25	1.75	1.68	1.17	1.18	1.71	1.67	1.32	1.25	1.86	1.78		
Condition day 0	-	-	1.26	1.26	-	1.23	1.22	1.22	1.23	1.22	1.23	1.24	1.23	1.22	1.23	1.20		
Condition day 58	-	-	1.35	1.33	-	1.09	1.30	1.29	1.06	1.06	1.26	1.27	1.07	1.06	1.33	1.29		

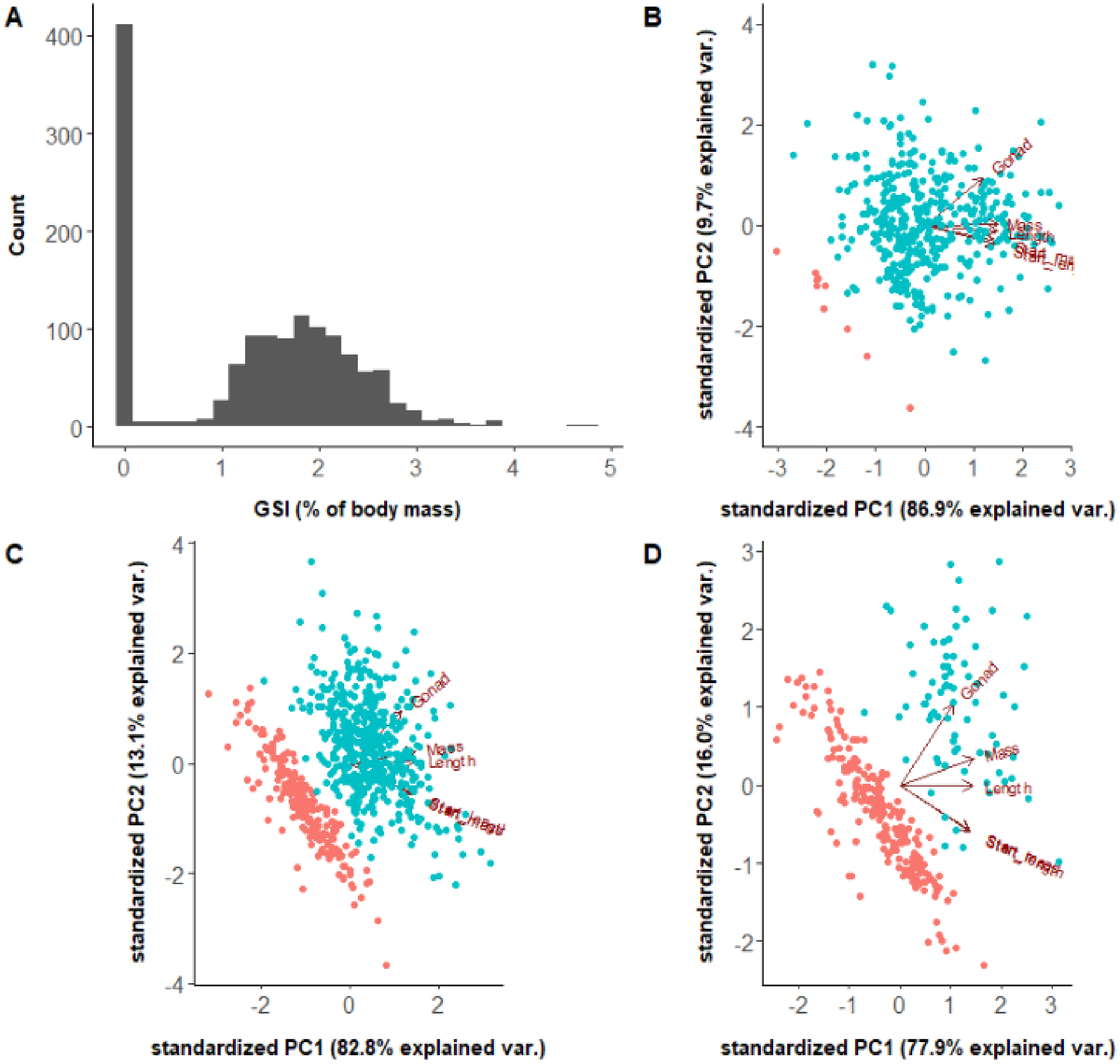

Supplementary Figure 1. Histogram of testis size (A). PCA plots for EE fish (A), EL fish (B), and LL fish (C) showing that the GSI cut-off of 0.2 leads to the same clustering as when accounting for growth.
